# Tumor Mutation Burden Survey of AACR GENIE Database Revealed NTRK ( *NTRK* +) and RET ( *RET* +) Fusions Positive Colorectal Carcinoma (CRC) as Distinct Subsets

**DOI:** 10.1002/cam4.70665

**Published:** 2025-02-14

**Authors:** Zhaohui Liao Arter, Alexandria T. M. Lee, Misako Nagasaka, Sai‐Hong Ignatius Ou

**Affiliations:** ^1^ Department of Medicine, Division of Hematology—Oncology University of California Irvine School of Medicine Orange California USA; ^2^ Chao Family Comprehensive Cancer Center Orange California USA

**Keywords:** colorectal cancer (CRC), *NRTK*, *RET*, TKI, tumor mutation burden (TMB)

## Abstract

**Background:**

Receptor tyrosine kinase (RTK) inhibitors have been approved for the treatment of NTRK fusion (*NTRK*+) and RET fusion (*RET*+) positive solid tumors in a tumor‐agnostic manner. However, the objective response rate was the lowest among entrectinib‐treated *NTRK*+ colorectal cancer (CRC) (20%) and selpercatinib‐treated *RET*+ CRC (20%) among all *NTRK*+, and *RET*+ solid tumors, respectively.

**Methods:**

We compared tumor mutation burden (TMB) in *NTRK+/RET+* CRC with all other *NTRK+* and *RET+* solid tumors using the American Association for Cancer Research (AACR) GENIE database (Version 13.0).

**Results:**

We identified 14,812 unique CRC patients. Considering only samples with identified fusion partners, the mean TMB was 66.6 ± 15.8 (mt/MB) for *NTRK+* CRC (*N* = 9) and 35 ± 11.5 for *RET+* CRC (*N* = 4), which were significantly higher when compared to the mean number of 6.2 ± 5.4 of TMB for all other RTK+ CRC (*N* = 30, *p* < 0.05). Furthermore, *NTRK*+ CRC harbored significantly higher TMB than *RET*+ CRC (*p* = 0.003). In comparison, the mean TMB was 4.0 ± 1.9 for *RET+* NSCLC (*N* = 65) and 2.6 ± 1.6 for *RET+* Thyroid cancer (*N* = 52). Mean TMB for all other *NTRK*+ solid tumors was < 11 and significantly lower than the mean TMB of *NTRK+* CRC. 1482 (10.0%) CRC patients had their MSI status reported. Three out of three *NTRK*+ CRC patients with known MSI status were all dMMR (100%). 0 out of 12 non‐*NTRK*/non‐*RET* RTK+ CRC patients were dMMR (0%).

**Conclusions:**

*NRTK+* and *RET+* CRC possess significantly higher TMB than other *RTK+* CRC or *NTRK+/RET+* non‐CRC solid tumors. TMB testing should be routinely done in MSI‐H CRC, and TMB ≥ 35 mut/MB samples should be screened for *NTRK* and *RET* fusions as an enrichment strategy to provide additional treatment for *NTRK+* and *RET+* CRC patients.

## Graphical abstract

*NRTK+* and *RET+* CRC possess significantly higher TMB than other *RTK+* CRC or *NTRK+/RET+* non‐CRC solid tumors. TMB testing should be routinely done in MSI‐H CRC and where TMB  > 35 mut/MB samples should be screened for *NTRK* and *RET* fusions as an enrichment strategy to provide an additional treatment option for *NTRK+* and *RET+* CRC patients.

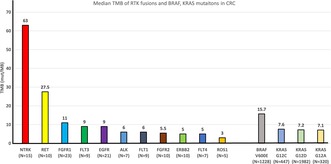

**Abbreviations**
AACR: American Association for Cancer Research
ALK: Anaplastic Lymphoma Kinase
CI: Confidence Interval
CRC: Colorectal Adenocarcinoma
dMMR: DNA Mismatch Repair deficient
DOR: Duration of Response
FDA: Food and Drug Administration
IO: Immune Oncology
KRAS: Kirsten Rat Sarcoma Viral Oncogene Homolog
MSI: Microsatellite Instability
MSI‐H: Microsatellite Instability‐High
NCCN: National Comprehensive Cancer Network
NGS: Next‐Generation Sequencing
NSCLC: Non‐Small Cell Lung Cancer
NTRK: Neurotrophic Tropomyosin Receptor Kinase
ORR: Overall Response Rate
PFS: Progression‐Free Survival
RET: Rearranged During Transfection
ROS1: c‐proto‐oncogene ROS1
RTK: Receptor Tyrosine Kinase
TKI: Tyrosine Kinase Inhibitor
TMB: Tumor Mutation Burden

## 1 Introduction

There are 58 human RTKs, which are classified into 20 distinct sub‐families based on their structural motifs, ligand specificities, and functional characteristics [1]. It is now well established that chromosomal rearrangement leading to fusions in several of these RTKs, though relatively rare, are actionable driver mutations in a wide range of solid malignancies [2, 3]. Tyrosine kinase inhibitors (TKIs) against prototypic RTK fusions such as anaplastic lymphoma kinase (*ALK*), c‐proto‐oncogene ROS1 (*ROS1*), rearranged during transfection (*RET*), and neurotrophic tropomyosin receptor kinases (*NTRK*) have received Food and Drug Administration (FDA) approval. Importantly, larotrectinib [4, 5] and entrectinib [6], both NTRK inhibitors, and selpercatinib [7], a RET TKI, have achieved tumor tissue‐agnostic indications against their respective targeted RTKs. While the efficacy of these three TKIs including overall response rate (ORR), duration of response (DOR), and median progression‐free survival (PFS) is generally reported in aggregate, upon closer examination, the ORR varies significantly by tumor types especially when treated with entrectinib for *NTRK*+ tumors and selpercatinib for *RET*+ tumors with the lowest ORRs reported among both *NTRK*+ [8] and *RET*+ colorectal adenocarcinoma (CRC) [9] (summarized in Table S1). The ORR among entrectinib‐treated *NTRK*+ CRC (20%, 2/10) was the lowest compared to the ORR of 61% for all other *N* *TRK* fusion positive solid tumors [8]. Similarly, among selpercatinib treated *RET*+ CRC patients, the ORR was (20%, 2/10) markedly lower compared to the ORR of 69% in patients with *RET* fusion‐positive non‐small cell lung cancer(NSCLC) and 79% in patients with *RET* fusion‐positive medullary thyroid cancer, as reported in the LIBRETTO‐001 trial [9]. Pralsetinib is another approved RET TKI for *RET* fusion‐positive NSCLC and thyroid cancer. The ARROW trial enrolled 29 patients with *RET*+ solid tumors other than NSCLC and thyroid cancer. While the ORR for the whole group (*N* = 23 evaluable patients) was 57% (95% CI: 35–77), none of the two *RET*+ CRC patients responded to pralsetinib [10].

It has been reported that both *NTRK+* [11] and *RET+* CRC [12] have high TMB with the median TMB of *NTRK+* CRC at 53 mut/MB [11] and the median TMB of *RET*+ CRC at 20 mut/MB [12]. Given the limited number of patients analyzed from previous commercial genomic sequencing databases, in this study, we investigated the differences between *NTRK*+/*RET*+ CRC and other *RTK+* CRC. We also compared the differences between *NTRK*+ CRC and other *NTRK*+ solid tumors, as well as between *RET*+ CRC and other *RET*+ solid tumors. This analysis utilized the publicly available AACR GENIE database to search for potential differences in ORR to TKIs in a tissue‐agnostic approach. Furthermore, we analyzed differences among *NTRK+/RET+* CRC, *BRAF* V600E+ and Kirsten Rat Sarcoma Viral Oncogene Homolog (*KRAS*) + CRC.

## 2 Materials and Methods

### 2.1 American Association for Cancer Research (AACR) Project Genomics Evidence Neoplasia Information Exchange (GENIE) Database

The American Association for Cancer Research (AACR) Project Genomics Evidence Neoplasia Information Exchange (GENIE) is an ongoing international pan‐cancer registry that has accumulated data from more than 110,000 tumors by 2022, making it the largest repository of publicly accessible, clinically annotated genomic data. We accessed GENIE version 13.0's patient data, which includes 148,222 patients and 167,358 samples [13]. It was publicly released in January 2023 and was accessed in March 2023. In accordance with 45 CFR 46, it was determined that the present study was exempt from institutional review board review and the requirement for informed consent because it utilized publicly available deidentified data. This report follows the Strengthening the Reporting of Observational Studies in Epidemiology (STROBE) reporting guidelines for retrospective cross‐sectional studies.

We first surveyed all 58 human RTKs to identify all RTK fusions in CRC. Second, we identified *RET* and *NTRK* fusions in all other solid tumors in addition to CRC, including non‐small cell lung cancer (NSCLC), thyroid cancer, breast cancer, pancreatic cancer, melanoma, prostate cancer, and ovarian cancer. We also identified cases with *BRAF* V600E, *KRAS* G12A/G12C/G12D mutations in CRC. We defined fusions as per AACR GENIE (the presence of a fusion partner or presence of intragenic rearrangement of the RTK). We extracted variables of interest from the database, including demographic data (age at sequencing, sex, and race), data source, tumor characteristics, and genomic alterations of each patient case. Genomic alterations information included the number and type of somatic mutations, structural variants, and copy number alterations.

### 2.2 Tumor Mutation Burden (TMB)

Tumor mutation burden (TMB) is not publicly listed but was able to be obtained through support from the AACR GENIE database statisticians. The methods of how AACR GENIC generated TMB have been previously reported [14]. Microsatellite instability (MSI)/Mismatch Repair deficient (dMMR) status was available through Genie BPC CRC v2.0‐public.

### 2.3 Statistical Analysis

Data were analyzed from January to April 2023. For continuous variable group comparisons, a 2‐sample *t* test was used. Two‐sided *p* < 0.05 was considered statistically significant.

## 3 Results

### 3.1 Distribution of RTK Fusions in CRC

**FIGURE 1:**
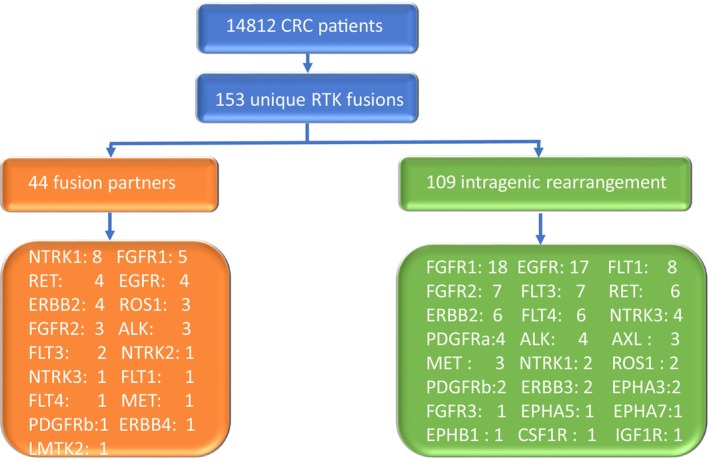
Distribution of the CRC patients analyzed in this study. CRC, colorectal cancer; RTK, Receptor tyrosine kinase.

**FIGURE 2:**
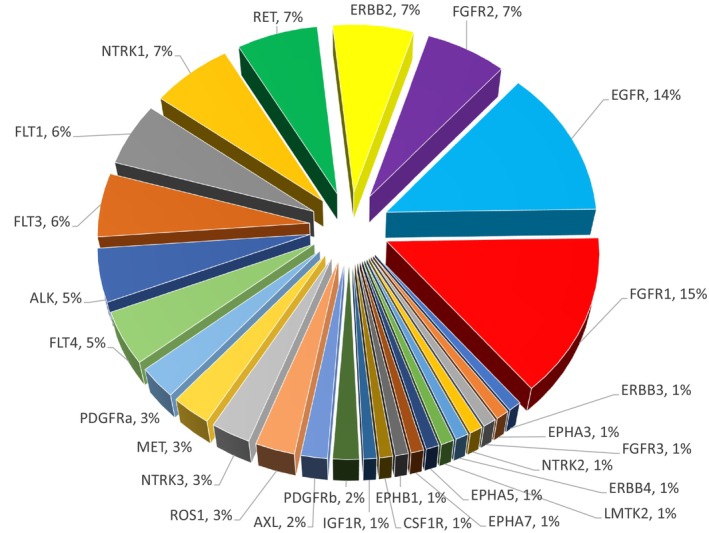
Pie chart distribution of all the RTK fusions identified in CRC patients in the AACR GENIE database. CRC, colorectal cancer; RTK, receptor tyrosine kinase.

Among the 14,812 unique CRC patients (57.8% colon adenocarcinoma, 22.1% colorectal adenocarcinoma, 16.9% rectal adenocarcinoma, 2.3% mucinous CRC) identified in GENIE version 13.0, a total of 153 unique RTK fusions were identified (44 with known fusion partners, 109 with intragenic rearrangement) in 27 out of the total 58 human RTKs (Figure 1). The most common alterations were in *FGFR1* (*N* = 23), *EGFR* (*N* = 21), *ERBB2* (*N* = 10), *NTRK1* (*N* = 10), *RET* (*N* = 10), *FGFR2* (*N* = 10), *FLT1* (*N* = 9), *FLT3* (*N* = 9), *FLT4* (*N* = 7), and *ALK* (*N* = 7). The proportion of RTK fusions identified in CRC is depicted in Figure 2.

### 3.2 Prevalence of RET Fusions in Solid Tumor Malignancies

Combining both fusion partner and intragenic fusions, we identified a total of 10 *RET+* CRC patients out of 14,812 CRC patients (0.07%), 109 *RET+* NSCLC patients out of 21,397 NSCLC patients (0.5%), and 75 *RET+* thyroid cancer patients out of 1944 non‐medullary thyroid cancer patients (3.9%). The results are consistent with a previous report that *RET* fusions are found primarily in NSCLC, thyroid, and CRC [12].

### 3.3 Prevalence of NTRK Fusions in Solid Tumors

For *NTRK1/3* fusions, we identified 15 *NTRK+* CRC cases (0.1%), 24 *NTRK+* NSCLC cases (0.1%), 28 *NTRK+* out of 2527 thyroid cancer cases (1.1%), 28 *NTRK+* out of 14,622 breast cancer patients (0.2%), 31 *NTRK+* out of 9392 glioma patients (0.3%), 10 *NTRK+* out of 6627 pancreatic cancer patients (0.2%), 14 *NTRK+* out of 6446 melanoma patients (0.2%), 7 *NTRK+* out of 5198 prostate cancer patients (0.1%), and 5 *NTRK+* out of 5900 ovarian cancer patients (0.08%). Overall consistent with literature, *NTRK* fusions are extremely rare [15, 16]. Detailed patient characteristics are presented in Table 1.

**TABLE 1:** Patient characteristics of the RET and NTRK fusion in CRC and other tumor types.

	RET			NTRK								
	*RET*+ CRC	*RET*+ NSCLC	*RET*+ non‐medullary Thyroid	*NTRK*+ CRC	*NTRK*+ NSCLC	*NTRK*+ Thyroid	*NTRK*+ Breast	*NTRK*+ Glioma	*NTRK*+ Pancreas	*NTRK*+ Melanoma	*NTRK*+ Prostate	*NTRK*+ Ovarian
*N*	10	109	75	15	24	28	28	31	10	14	7	5
Age at sequencing												
Mean (SD)	71.3 (8.4)	61 (13.8)	41.3 (21.2)	63.6 (9.3)	55.2 (17.4)	39.3 (22.8)	47.4 (15.3)	39.8 (20.1)	61.6 (9.8)	48.1 (22.9)	68.4 (8.8)	61.2 (6.9)
Range	59–88	25–> 89^a^	< 18–88^a^	45–84	23–> 89^a^	< 18–82^a^	< 18–72^a^	< 18–72^a^	39–72	17–76	55–79	54–71
Sex												
Female	7 (70%)	52 (55.9%)	49 (71%)	11 (73.3%)	13 (56.5%)	18 (66.7%)	24 (92.3%)	12 (41.4%)	5 (50%)	8 (57.1%)	0	5 (100%)
Male	3 (30%)	41 (44.1%)	20 (29%)	4 (26.7%)	10 (43.5%)	9 (33.3%)	2 (7.7%)	17 (58.6%)	5 (50%)	5 (35.7%)	7 (100%)	0
Unknown										1 (7.1%)		
Race												
Non‐Hispanic White	9 (90%)	67 (72.5%)	37 (53.6%)	12 (80%)	19 (82.6%)	18 (66.7%)	16 (61.5%)	14 (48.3%)	9 (90%)	9 (64.3%)	7 (100%)	4 (80%)
Asian	0	14 (15.1%)	7 (10.1%)	0	1 (4.3%)	3 (11.1%)	0	1 (3.4%)	1 (10%)	0	0	0
Hispanic	0	6 (6.5%)	10 (14.5%)	1 (6.7%)	1 (4.3%)	2 (7.4%)	2 (7.7%)	1 (3.4%)	0	0	0	0
Non‐Hispanic Black	1 (10%)	3 (3.2%)	4 (5.8%)	1 (6.7%)	1 (4.3%)	2 (7.4%)	4 (15.4%)	0	0	0	0	0
Native American	0	0	0	0	1 (4.3%)	0	0	0	0	0	0	0
Pacific Islander	0	0	0	0	0	0	0	0	0	0	0	1 (20%)
Unknown	0	3 (3.3%)	11 (15.9%)	1 (6.7%)	0	2 (7.4%)	4 (15.4%)	13 (44.8%)	0	5 (35.7%)	0	0
Fusion												
Partner	4	65	52	9	7	20	7	21	2	8	1	0
Intragenic	6	44	23	6	17	8	21	10	8	6	6	5
TMB												
Mean (SD)	26.6 (21.7)	4.4 (3.8)	2.5 (1.5)	55.1 (25.5)	10.5 (10.8)	3.5 (2.6)	4.6 (4.2)	3.0 (1.8)	3.5 (1.8)	25.3 (42.4)	3.9 (2.9)	6.4 (3.4)
Range	1.7–60.6	0.5–29	0.5–8.7	3.2–95	0.9–39.5	0.7–14.5	0.9–21.4	0.5–8.8	1.4–6.8	1.9–154	0.9 to 9.2	2.3–10.2

Abbreviations: CRC, colorectal cancer; NSCLC, non‐small cell lung cancer; SD, standard deviation; TMB, tumor mutation burden.

^a^ 90 was used for calculation when age is > 89 and 17 was used for calculation when age is < 18.

### 3.4 Number of Genomic Co‐Alterations

The mean number of genomic co‐alterations was 69.9 ± 13.2 for *NTRK1+* CRC with identified fusion partner (*N* = 8), 72.3 ± 24.1 co‐mutations for *RET+ CRC* identified fusion partner (*N* = 4). Including intragenic rearrangements, the mean number was 67.7 ± 13.2 for *NTRK1+* CRC (*N* = 10) and 43.6 ± 36.4 for *RET+* CRC (*N* = 10).

Combining *NTRK1+/NTRK3*+/*RET*+ CRC with known fusion partners (*N* = 13), the mean number of genomic co‐mutations was 68.3 ± 7.9 compared to the mean number of 11.8 ± 11.1 co‐mutations for all other *RTK+* CRC (*ALK*, *EGFR*, *FGFR1/2*, *ERBB4*, *FLT1/3/4*, *LMKT2*, *MET*, *NTRK2*, *PDGFR‐β*, *ROS1*) with known fusion partners (*N* = 30, one sample with no recorded genomic co‐mutations, one duplicated sample).

**FIGURE 3:**
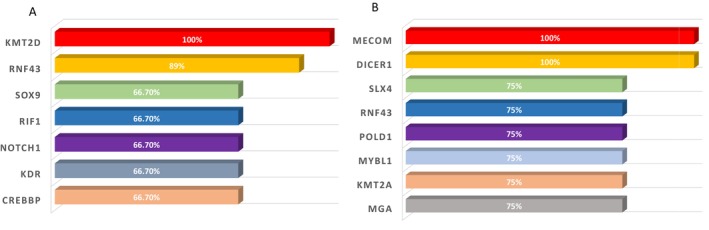
Bar chart of the major co‐genomic alterations among *NTRK*+ (A) and *RET*+ CRC (B).

Among *NTRK1/3* fusion partner cases (*N* = 9), the most common co‐alterations were *KMT2D* (9/9, 100%), *RNF43* (8/9, 88.9%), *CREBBP* (6/9, 66.7%), *KDR* (6/9, 66.7%), *NOTCH1* (6/9, 66.7%), *RIF1* (6/9, 66.7%), and *SOX9* (6/9, 66.7%) (Figure 3A, Table 2A). *POLD1* or *POLE* was detected in five out of nine patients (55.6%) consistent with findings from a previous report [11]. *BRAF* mutation was identified in two out of nine patients; however, no *KRAS* or *NRAS* mutations were detected in *NTRK*+ CRC cases. Among *RET* fusion partner cases (*N* = 4), the most common co‐alterations were *DICER1* (4/4, 100%), *MECOM* (4/4, 100%), *MGA* (3/4, 75%), *KMT2A* (3/4, 75%), *MYBL1* (3/4, 75%), *POLD1*(3/4, 75%), *RNF43* (3/4, 75%), and *SLX4* (3/4, 75%) (Figure 3B, Table 2B). *BRAF* mutations were identified in one out of four patients, whereas neither *KRAS* nor *NRAS* mutations were detected in *RET*+ CRC cases.

**TABLE 2:** Classification of major co‐genomic alterations among *NTRK*+ (A) and *RET*+ CRC(B).

Gene	Classification	Function description
A		
KMT2D	Chromatin modifying gene	Histone methyltransferase modifying H3K4, regulating transcription and epigenetics
RNF43	Ubiquitin ligase	E3 ubiquitin‐protein ligase regulating Wnt signaling
SOX9	Transcription factor	Critical in chondrogenesis, sex determination, and developmental gene regulation
RIF1	DNA repair and replication	Regulates DNA replication timing and repair, involved in DNA damage response
NOTCH1	Signaling pathway component	Notch signaling receptor regulating cell fate, proliferation, and apoptosis
KDR	Receptor tyrosine kinase	Receptor for VEGFs, key role in angiogenesis and endothelial cell proliferation
CREBBP	Chromatin modifying/co‐activator	Histone acetyltransferase, co‐activator in transcription regulation

B		
MECOM	Transcription factor/oncogene	Regulates hematopoiesis, cell proliferation, and differentiation; role in leukemia
DICER1	RNA processing enzyme	Processes miRNA and siRNA, involved in RNA interference and gene silencing
SLX4	DNA repair	Scaffold protein in DNA repair, resolves Holliday junctions and interstrand crosslinks
RNF43	Ubiquitin ligase	Regulates Wnt signaling by ubiquitinating Frizzled receptors
POLD1	DNA replication and repair	Catalytic subunit of DNA polymerase delta, crucial for DNA replication and repair
MYBL1	Transcription factor	Regulates gene expression during cell proliferation, differentiation, and apoptosis
KMT2A	Chromatin modifying gene	Histone methyltransferase for H3K4, regulates gene expression and development
MGA	Transcription factor	Regulates MYC target genes, involved in transcriptional repression and differentiation

### 3.5 Tumor Mutation Burden (TMB)

Considering only samples with identified fusion partners, the mean TMB was 66.6 ± 15.8 (mt/MB) for *NTRK1/3*+ CRC (*N* = 9), and 35 ± 11.5 for *RET*+ CRC (*N* = 4), which were significantly higher when compared to the mean number of 6.2 ± 5.4 of TMB for all other *RTK+* CRC (*ALK*, *EGFR*, *FGFR1/2*, *ERBB4*, *FLT1/3/4*, *LMKT2*, *MET*, *NTRK2*, *PDGFR‐b*, *ROS1*) (*N* = 30, *p* < 0.05). Furthermore, *NTRK1/3*+ CRC harbored significantly higher TMB than *RET*+ CRC (*p* = 0.003).

**FIGURE 4:**
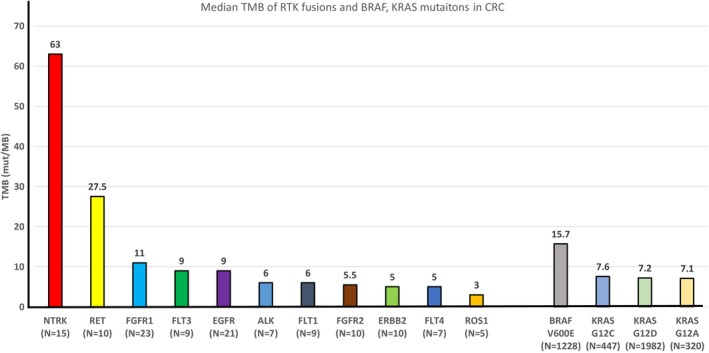
Bar chart graph of TMB of RTK fusions and *BRAF* V600E, *KRAS* G12A/C/D mutations in CRC. CRC, colorectal cancer; TMB, tumor mutation burden; RTK, receptor tyrosine kinase.

Considering both fusion partners and intra‐ and inter‐chromosomal rearrangement, the mean TMB was 55.1 ± 25.5 for *NTRK1/3+* CRC (*N* = 15), and 26.6 ± 21.7 for *RET+* CRC (*N* = 10), which were significantly higher when compared to other RTK+ CRC (*p* < 0.05) (Table 1). The median TMB was 63 for *NTRK+* CRC (*N* = 15) (95% CI: 4–82), and 27.5 for *RET*+ CRC (*N* = 10) (95% CI: 4–49), which were also higher when compared to other *RTK+* CRC (Figure 4).

The mean TMB was 4.0 ± 1.9 for *RET*+ NSCLC (*N* = 65), 2.6 ± 1.6 for *RET*+ thyroid cancer (*N* = 52), both of which were significantly lower than *RET*+ CRC. Mean TMB was 3.1 ± 1.7 for *NTRK*+ glioma (*N* = 21), 3.3 ± 1.7 for *NTRK*+ thyroid cancer (*N* = 21), 6.5 ± 5.1 for *NTRK*+ melanoma (*N* = 8), 5.9 ± 3.4 for *NTRK*+ NSCLC (*N* = 7), and 5.1 ± 1.2 for *NTRK*+ breast cancer (*N* = 7). All of them were significantly lower than the mean TMB of *NTRK*+ CRC (Table 1).

### 3.6 *BRAF* V600E+ CRC and *KRAS* G12D/C/A+ CRC

A total of 1228 (8.3%) *BRAF* V600E mutant CRC cases were identified with a median TMB of 15.7 (95% CI: 0–225). In addition, there were 1982 (13.4%) cases of *KRAS* G12D, 447 (3.0%) cases of *KRAS* G12C, and 320 (2.2%) cases of *KRAS* G12A with median TMBs of 7.2 (95% CI: 0–224), 7.6 (95% CI: 0–260), and 7.1 (95% CI: 0–225), respectively (Figure 4). The mean TMB values were 46.2 ± 72.9 for *BRAF* V600E, 53.6 ± 367.5 for *KRAS* G12D, 81.2 ± 389.6 for *KRAS* G12C, and 55.8 ± 275.6 for *KRAS* G12A (Figure S1). In CRC with *BRAF* V600E, 523 (42.6%) had TMB ≥ 35, 382 (31.1%) had TMB ≥ 50, 245 (20%) had TMB ≥ 75, and 149 (12.1%) had TMB ≥ 100. In CRC with *KRAS* G12D, 462 (23.3%) had TMB ≥ 35, 406 (20.5%) had TMB ≥ 50, 311 (15.7%) had TMB ≥ 75, and 241 (12.2%) had TMB ≥ 100. In CRC with *KRAS* G12C, 115 (25.7%) had TMB ≥ 35, 103 (23%) had TMB ≥ 50, 93 (20.8%) had TMB ≥ 75, and 65 (14.5%) had TMB ≥ 100. In CRC with *KRAS* G12A, 74 (23.1%) had TMB ≥ 35, 68 (21.3%) had TMB ≥ 50, 62 (19.4%) had TMB ≥ 75, and 48 (15%) had TMB ≥ 100.

### 3.7 Microsatellite Instability Status

MSI status was available in 1482 out of 14,812 cases (10%) of CRC. Three out of three *NTRK*+ CRC patients with known MSI status were all dMMR (100%). Among the *RET*+ CRC cases (*N* = 10) (partners + intragenic rearrangement), none of the MSI or MMR status were available. Zero of 12 non‐*NTRK*/non‐*RET* RTK+ CRC patients were dMMR (0%) (Table S2).

In *BRAF* V600E mutated CRC, 102 individuals (8.3%) had MMR/MSI status reported. Among these, 38 patients (38%) were identified with dMMR/MSI‐H. In the microsatellite stable group, the mean TMB was 11 ± 18.4, and the median TMB was 6. Conversely, in the MSI‐H group, the mean TMB was 56.1 ± 19.7, and the median TMB was 44. A statistically significant difference in TMB was observed between these two groups (*p* < 0.01). Among 162 patients with the *KRAS* G12D mutation (8.2% of the cohort), 12 (7.4%) were found to have dMMR/MSI‐H. In contrast, none of the 34 patients with the *KRAS* G12C mutation (7.6% reported) and none of the 21 patients with the *KRAS* G12A mutation (6.6% reported) showed evidence of dMMR/MSI‐H.

## 4 Discussion

Tissue‐agnostic indication and approval for oncology treatment based on a common molecular alteration, which implies a shared pathogenesis pathway, is one of the greatest achievements in converging cancer science and oncology drug development. This achievement should be celebrated and encouraged. Tumor‐specific response analysis of NTRK and RET TKIs across various *NTRK+* and *RET+* solid tumors indicated a heterogeneous response with the poorest outcomes observed in CRC. *NTRK* and *RET* fusions are exceedingly rare in CRC. Gouda MA et al. [13] reported 29 *NTRK*+ (1.7%) and 15 *RET+* CRC (0.9%) samples using the GENIE database, exceeding the numbers identified in our study. Their analysis utilized sample‐based reporting, whereas our study focused on patient‐level data to avoid duplication from multiple samples derived from the same individual. Furthermore, our analysis was restricted to typical CRC subtypes, whereas their study may have included atypical subtypes, potentially contributing to the observed differences. Our analysis of the AACR GENIE database of these extremely rare *NTRK+* and *RET+* CRC indicates that DNA mismatch repair leading to high TMB may underpin the generation of only *NTRK* and *RET* fusions in CRC, and that *NTRK* and *RET* fusions in CRC appear to act more as secondary passenger mutations rather than primary driver mutations.

*NTRK+* CRC with known fusion partners harbored a mean TMB of 66.6 mut/MB, and a mean of 53.4 mut/MB for all *NTRK* fusions defined by GENIE, while all other types of solid tumors with *NTRK* fusions were found to have a much lower mean TMB of < 11 mut/MB (range 3–10.5 mut/MB). *RET+* CRC with identified fusion partners was also found to have a significantly higher TMB when compared to *RET*+ NSCLC and *RET*+ thyroid cancer. Furthermore, *NTRK+* CRC consistently harbored the highest TMB among other rare RTK fusions identified in CRC.

With a limited number of data, many of these high TMB in *NTRK*+ CRC are also MSI‐H (MMR deficient), which likely explains the high number of genomic co‐alterations and may explain the *NTRK* fusion event. *NTRK*+ CRC were more likely to be MSI‐H (100%) when compared to non‐*NTRK*/non‐*RET* RTK+ CRC patients (0%). Wang et al. [11] reported that *NTRK*‐driven colorectal cancer patients exhibited increased TMB (median = 53 mut/MB, 95% CI: 36.8–68.0 mut/MB) and were frequently MSI‐H. Similarly, Nagasaka et al. [12] found that *RET*‐positive CRC harbored higher median TMB and were commonly MSI‐H. Our findings are consistent with the results of Nagasaka et al. In Nagasaka's paper, the Caris Life Science database was utilized. However, the primary contributors to the GENIE database, including DFCI, MSKCC, UCSF, and Johns Hopkins, do not routinely use Caris, likely accounting for the discrepancy in the number of *RET*‐positive CRC cases. We hypothesize that *NTRK* fusion (and *RET* fusion) may not be the primary driver alterations in *NTRK*+ NSCLC but may act as a secondary event due to DNA mismatch repair deficiency and thus may explain the lower ORR observed with NTRK TKIs and RET TKI in *NTRK+* and *RET+* CRC, respectively. Nonetheless, why MSI‐H or high TMB status did not result in the generation of other RTK fusions in CRC but only in *NTRK* and *RET* fusions is unknown. Given the rarity of these fusions, large‐scale database studies may offer valuable insights.

Immune checkpoint inhibitor pembrolizumab, dostarlimab, or nivolumab with ipilimumab have been approved for patients with MSI‐H/dMMR CRC, with pembrolizumab or dostarlimab approved for all MSI‐H/dMMR regardless of tissue origin [15, 16, 17]. Hence, the current National Comprehensive Cancer Network (NCCN) suggests MSI testing for all metastatic CRC and gives immunotherapy (IO) alone if MSI‐H.

**FIGURE 5:**
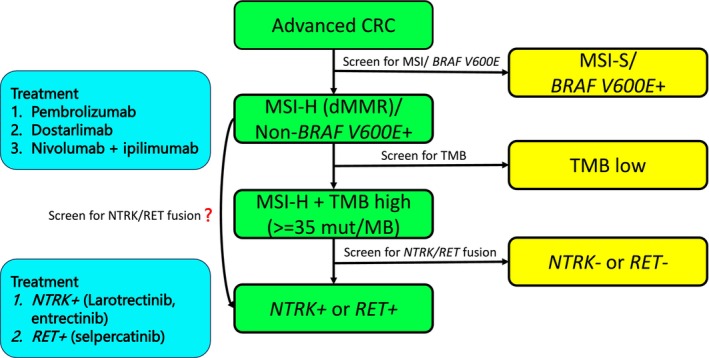
Potential screening algorithm for MSI‐H CRC patients. CRC, colorectal cancer; dMMR, mismatch repair deficient; MSI‐H, microsatellite instability–high; MSI‐S, microsatellite instability–stable; TMB, tumor mutation burden.

TMB measures the total amount of somatic coding mutations within a given coding area of the tumor genome. It is believed that highly mutated tumors harbor an increased neoantigen burden, rendering them immunogenic and amenable to immunotherapy. Pembrolizumab has been approved for TMB‐H tumors regardless of tumor origin by the US FDA since June 2020 based on KEYNOTE‐158 [18]. In a subsequent retrospective analysis of 137 patients treated with pembrolizumab, however, the benefit was limited to those with high TMB and either dMMR or pol‐deficient (*POLE* or *POLD1* pathogenic mutations) [19]. TMB in MSI‐H mCRC is generally elevated but can be variable [20]. Approximately 3%–5% of patients with metastatic CRCs and proficient mismatch repair (pMMR) have high TMB levels [20, 21, 22]. The NCCN Panel does not currently recommend TMB testing in metastatic CRC as part of routine clinical care, although this will identify additional CRC patients eligible for IO therapy. Furthermore, our findings indicate that MSI‐H patients with TMB > 35 mut/MB have an increased chance of harboring an *NTRK* or *RET* fusion and thus may benefit from *NRTK* or RET TKIs, respectively. Given the rarity of *NTRK*+ or *RET*+ CRC or even general RTK fusions in CRC, clinicians may not routinely screen for RTK fusions given the technical challenges involved. Optimal screening for RTK fusions is best achieved using RNA next‐generation sequencing (NGS) [23], which may not be readily accessible or widely implemented. Thus, sequential testing for MSI‐H and TMB is a strategy to enrich detection of *NTRK* and *RET* fusions in CRC (Figure 5). Finally, the median TMB of *BRAF* V600E+ CRC is relatively high, with the mean TMB closely approximating that observed in *NTRK+/RET+* CRC. Limited data showed a substantial proportion of these patients (38%) exhibit dMMR or MSI‐H, indicating there is a unique subset of *BRAF* V600E+ CRC. Encorafenib and cetuximab have been approved for the treatment of *BRAF* V600E+ CRC based on the BEACON‐CRC trial [24]. The addition of pembrolizumab to encorafenib and cetuximab is being investigated in *BRAF* V600E/MSI‐H CRC [25]. Similarly, the median TMB for *KRAS* G12D/C/A+ CRC was low in the single digits, whereas the mean was high, indicating that a subset of samples exhibited very high TMB as defined by the GENIE database. The combination of the KRAS inhibitor sotorasib and the EGFR antibody panitumumab has demonstrated activity in chemotherapy‐refractory *KRAS* G12C+ CRC [26]. Given our finding, determining TMB in *KRAS* G12+ CRC may support the addition of IO to the combination treatment regimen.

This study has several limitations. First, the genomic profiling platforms are not uniform, as multiple institutions contributing to the databases use different sequencing platforms. These platforms may not include RNA NGS, which is crucial for detecting in‐frame RTK fusions. Second, we defined RTK fusions as samples with either fusion partners or intra‐genic rearrangements. As mentioned earlier, without RNA NGS, some intragenic rearrangements may not translate into functional fusions. Therefore, we first reported TMB from samples with known fusion partners. Among the 15 *NTRK+* fusions, 10 had a fusion partner, and among the 10 *RET* fusions, 4 had a fusion partner. Third, the DNA NGS gene panels may vary from one contributing institution to another and hence, the number of genomic co‐alterations may be restricted by the number of genes sequenced. Similarly, the definition of TMB may differ between institutions. Nevertheless, considering the extreme rarity of these fusions in CRC, the AACR GENIE database represents the largest open public resource for investigators. Finally, stage and treatment history, and survival outcomes of CRC were not captured, and the data on MSI is limited to a minor subset of CRC samples.

## 5 Conclusion

This extensive cross‐sectional study of human RTK fusions in solid tumors revealed that *NRTK*+ and *RET*+ CRC possess significantly higher TMB than other *RTK+* CRC or *NTRK*+/*RET*+ non‐CRC solid tumors. *NTRK*+ and *RET*+ CRC are distinct molecular subtypes of CRC with distinct pathogenesis arising from deficient DNA mismatch repair in comparison to all other *NTRK*+ or *RET*+ solid tumors. Given the rarity of *NTRK* and *RET* fusions in solid tumors, a high TMB in MSI‐H CRC is an indication for screening. The underlying mechanism by which mismatch repair may generate only *NTRK* and *RET* fusions in CRC remains to be investigated. Finally, a subset of *BRAF* V600E+ and *KRAS* G12D/C/A+ CRC harbored high TMB that warrants further detailed investigation.

## Author Contributions

**Zhaohui Liao Arter:** conceptualization (equal), data curation (lead), formal analysis (lead), methodology (equal), writing – original draft (equal), writing – review and editing (equal). **Alexandria T. M. Lee:** writing – review and editing (equal). **Misako Nagasaka:** conceptualization (equal), supervision (equal), writing – review and editing (equal). **Sai‐Hong Ignatius Ou:** conceptualization (equal), methodology (equal), supervision (lead), writing – original draft (equal), writing – review and editing (equal).

## Ethics Statement

In accordance with 45 CFR 46, it was determined that the present study was exempt from institutional review board review and the requirement for informed consent because it utilized publicly available deidentified data. This report follows the Strengthening the Reporting of Observational Studies in Epidemiology (STROBE) reporting guidelines for retrospective cross‐sectional studies.

## Consent

This is an observational study involving the analysis of secondary data only. All data were deidentified. There was no direct interaction with human subjects for this study.

## Conflicts of Interest

Ou has received speaker bureau honoraria from Pfizer, Janssen/JNJ, DAVA Oncology LLP, and OncLive. He also holds advisory board roles with AnHeart Therapeutics, Pfizer, Janssen/JNJ, Daiichi Sankyo, BMS, and Elevation Oncology, for which he receives honoraria. Additionally, he serves on the scientific advisory boards for Elevation Oncology and AnHeart Therapeutics and has stock ownership in, Nuvalent, Nuvation, MBrace Therapeutics and BlossomHill Therapeutics. Nagasaka has received consulting fees from Caris Life Sciences, honoraria from AstraZeneca, Daiichi Sankyo, Novartis, Lilly, Pfizer, EMD Serono, Genentech, Regeneron, and BMS. She is a speaker for Mirati, Takeda, Janssen, and Blueprint Medicine and has received travel support from AnHeart Therapeutics.

## Supporting information


**Appendix S1.** Supporting Information.

## Data Availability

The data utilised in this research is publicly available except for Tumor mutation burden (TMB) which was obtained from the AACR GENIE database statistician.

## References

[1] P. Blume‐Jensen and T. Hunter , “Oncogenic Kinase Signaling,” Nature 411, no. 6835 (2001): 355–365, 10.1038/35077225. 11357143

[2] A. T. Shaw , P. P. Hsu , M. M. Awad , and J. A. Engelman , “Tyrosine Kinase Gene Rearrangements in Epithelial Malignancies,” Nature Reviews. Cancer 13, no. 11 (2013): 772–787, 10.1038/nrc3612. 24132104 PMC3902129

[3] A. M. Schram , M. T. Chang , P. Jonsson , and A. Drilon , “Fusions in Solid Tumours: Diagnostic Strategies, Targeted Therapy, and Acquired Resistance,” Nature Reviews. Clinical Oncology 14, no. 12 (2017): 735–748, 10.1038/nrclinonc.2017.127. PMC10452928 28857077

[4] A. Mullard , “FDA Approves Landmark Tissue‐Agnostic Cancer Drug,” Nature Reviews Drug Discovery 18, no. 1 (2018): 7–8, 10.1038/nrd.2018.226. 30591721

[5] D. Hong , S. BuBois , S. Kummar , et al., “Larotrectinib in Patients With TRK Fusion‐Positive Solid Tumours: A Pooled Analysis of Three Phase 1/2 Clinical Trials,” Lancet Oncology 21, no. 4 (2020): 531–540. 32105622 10.1016/S1470-2045(19)30856-3 PMC7497841

[6] L. Marcus , M. Donoghue , S. Aungst , et al., “FDA Approval Summary: Entrectinib for the Treatment of NTRK Gene Fusion Solid Tumors,” Clinical Cancer Research 27, no. 4 (2021): 928–932, 10.1158/1078-0432.CCR-20-2771. 32967940

[7] E. S. Duke , D. Bradford , M. Marcovitz , et al., “FDA Approval Summary: Selpercatinib for the Treatment of Advanced RET Fusion‐Positive Solid Tumors,” Clinical Cancer Research 29, no. 18 (2023): OF1–OF6, 10.1158/1078-0432.CCR-23-0459. PMC10524590 37265412

[8] G. D. Demetri , F. De Braud , A. Drilon , et al., “Updated Integrated Analysis of the Efficacy and Safety of Entrectinib in Patients With NTRK Fusion‐Positive Solid Tumors,” Clinical Cancer Research 28, no. 7 (2022): 1302–1312, 10.1158/1078-0432.CCR-21-3597. 35144967 PMC9365368

[9] V. Subbiah , J. Wolf , B. Konda , et al., “Tumour‐Agnostic Efficacy and Safety of Selpercatinib in Patients With RET Fusion‐Positive Solid Tumours Other Than Lung or Thyroid Tumours (LIBRETTO‐001): A Phase ½, Open‐Label, Basket Trial,” Lancet Oncology 23, no. 10 (2022): 1261–1273, 10.1016/S1470-2045(22)00541-1. 36108661 PMC11702314

[10] V. Subbiah , P. A. Cassier , S. Siena , et al., “Pan‐Cancer Efficacy of Pralsetinib in Patients With RET Fusion‐Positive Solid Tumors From the Phase ½ ARROW Trial,” Nature Medicine 28, no. 8 (2022): 1640–1645, 10.1038/s41591-022-01931-y. PMC9388374 35962206

[11] H. Wang , Z. W. Li , Q. Ou , et al., “NTRK Fusion Positive Colorectal Cancer Is a Unique Subset of CRC With High TMB and Microsatellite Instability,” Cancer Medicine 11, no. 13 (2022): 2541–2549, 10.1002/cam4.4561. 35506567 PMC9249987

[12] M. Nagasaka , D. Brazel , Y. Baca , et al., “Pan‐Tumor Survey of RET Fusions as Detected by Next‐Generation RNA Sequencing Identified RET Fusion Positive Colorectal Carcinoma as a Unique Molecular Subset,” Translational Oncology 36 (2023): 101744, 10.1016/j.tranon.2023.101744. 37516008 PMC10410168

[13] M. A. Gouda , B. E. Nelson , L. Buschhorn , A. Wahida , and V. Subbiah , “Tumor‐Agnostic Precision Medicine From the AACR GENIE Database: Clinical Implications,” Clinical Cancer Research 29, no. 15 (2023): 2753–2760, 10.1158/1078-0432.CCR-23-0090. 37061987 PMC10390861

[14] J. Anaya , J. W. Sidhom , C. A. Cummings , A. S. Baras , and AACR Project GENIE Consortium , “Probabilistic Mixture Models Improve Calibration of Panel‐Derived Tumor Mutational Burden in the Context of Both Tumor‐Normal and Tumor‐Only Sequencing,” Cancer Research Communications 3, no. 3 (2023): 501–509, 10.1158/2767-9764.CRC-22-0339. 36999044 PMC10044680

[15] T. André , S. Lonardi , K. Y. M. Wong , et al., “Nivolumab Plus Low‐Dose Ipilimumab in Previously Treated Patients With Microsatellite Instability‐High/Mismatch Repair‐Deficient Metastatic Colorectal Cancer: 4‐Year Follow‐Up From CheckMate 142,” Annals of Oncology 33, no. 10 (2022): 1052–1060, 10.1016/j.annonc.2022.06.008. 35764271

[16] D. Berton , S. Banerjee , G. Curigliano , et al., “Antitumor Activity of Dostarlimab in Patients With Mismatch Repair‐Deficient/Microsatellite Instability–High Tumors: A Combined Analysis of Two Cohorts in the GARNET Study,” Journal of Clinical Oncology 39, no. 15 (2021): 2564, 10.1200/JCO.2021.39.15_suppl.2564.34101481

[17] T. André , K. Shiu , T. Kim , et al., “Pembrolizumab in Microsatellite‐Instability‐High Advanced Colorectal Cancer,” New England Journal of Medicine 383, no. 23 (2020): 2207–2218, 10.1056/NEJMoa2017699. 33264544

[18] A. Marabelle , M. Fakih , J. Lopez , et al., “Association of Tumour Mutational Burden With Outcomes in Patients With Advanced Solid Tumours Treated With Pembrolizumab: Prospective Biomarker Analysis of the Multicohort, Open‐Label, Phase 2 KEYNOTE‐158 Study,” Lancet Oncology 21, no. 10 (2020): 1353–1365, 10.1016/S1470-2045(20)30445-9. 32919526

[19] B. Rousseau , M. B. Foote , S. B. Maron , et al., “The Spectrum of Benefit From Checkpoint Blockade in Hypermutated Tumors,” New England Journal of Medicine 384, no. 12 (2021): 1168–1170. 33761214 10.1056/NEJMc2031965 PMC8403269

[20] D. A. Fabrizio , T. J. George, Jr. , R. F. Dunne , et al., “Beyond Microsatellite Testing: Assessment of Tumor Mutational Burden Identifies Subsets of Colorectal Cancer Who May Respond to Immune Checkpoint Inhibition,” Journal of Gastrointestinal Oncology 9, no. 4 (2018): 610–617. 30151257 10.21037/jgo.2018.05.06 PMC6087857

[21] A. B. Schrock , C. E. Devoe , R. McWilliams , et al., “Genomic Profiling of Small‐Bowel Adenocarcinoma,” JAMA Oncology 3, no. 11 (2017): 1546–1553, 10.1001/jamaoncol.2017.1051. 28617917 PMC5710195

[22] A. R. Parikh , Y. He , T. S. Hong , et al., “Analysis of DNA Damage Response Gene Alterations and Tumor Mutational Burden Across 17,486 Tubular Gastrointestinal Carcinomas: Implications for Therapy,” Oncologist 24, no. 10 (2019): 1340–1347, 10.1634/theoncologist.2019-0034. 31040255 PMC6795150

[23] S. Yang , U. Aypar , E. Rosen , D. Mata , R. Benayed , and K. Mullaney , “A Performance Comparison of Commonly Used Assays to Detect RET Fusions,” Clinical Cancer Research 27, no. 5 (2021): 1316–1328. 33272981 10.1158/1078-0432.CCR-20-3208 PMC8285056

[24] S. Kopetz , A. Grothey , R. Yaeger , et al., “Encorafenib, Binimetinib, and Cetuximab in BRAF V600E‐Mutated Colorectal Cancer,” New England Journal of Medicine 381, no. 17 (2019): 1632–1643. 31566309 10.1056/NEJMoa1908075

[25] E. Elez , S. Kopetz , J. Tabernero , et al., “SEAMARK: Phase II Study of First‐Line Encorafenib and Cetuximab Plus Pembrolizumab for MSI‐H/dMMR BRAFV600E‐Mutant mCRC,” Future Oncology 20, no. 11 (2023): 653–663, 10.2217/fon-2022-1249. 37815847 PMC12510239

[26] M. G. Fakih , L. Salvatore , T. Esaki , et al., “Sotorasib Plus Panitumumab in Refractory Colorectal Cancer With Mutated KRAS G12C,” New England Journal of Medicine 389, no. 23 (2023): 2125–2139. 37870968 10.1056/NEJMoa2308795

