# Supplementary material for: Tumor Mutation Burden Survey of AACR GENIE Database Revealed NTRK ( NTRK +) and RET ( RET +) Fusions Positive Colorectal Carcinoma (CRC) as Distinct Subsets
Source: Cancer Med. 2025 Feb 14;14(4):e70665. doi: 10.1002/cam4.70665 (PMC11826831; doi:10.1002/cam4.70665)
Supplement: Supplementary file 1 — Appendix S1. Supporting Information. [file CAM4-14-e70665-s001.docx]

**Supplementary Appendix**

**Supplementary Tables:**

**Table S1:** ORR for NTRK+ /RET+ solid tumors


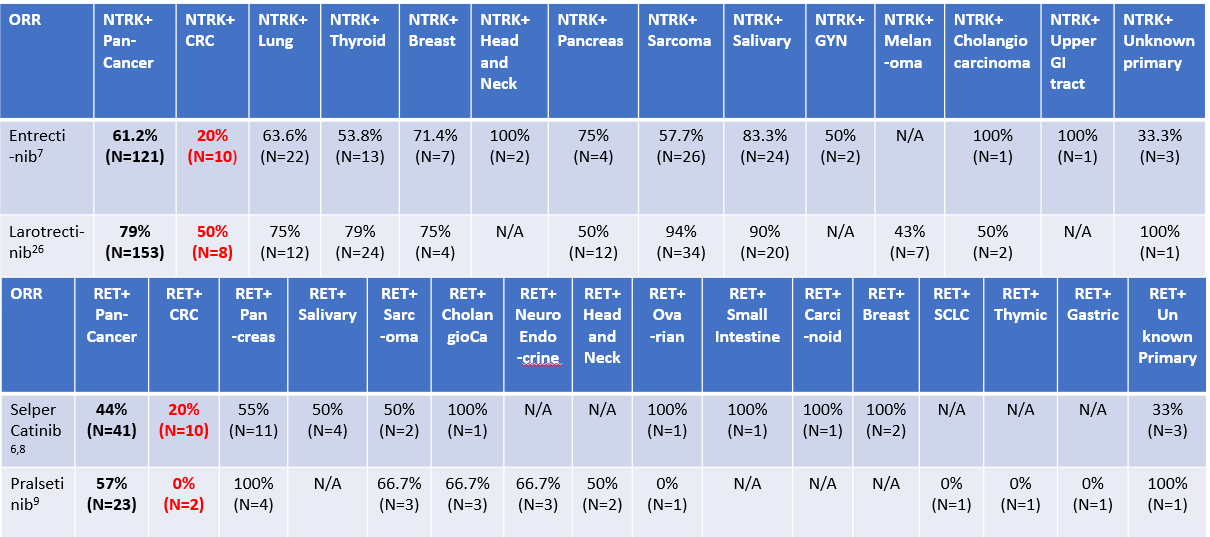


ORR: objective response rate

**Table S2:** MSI/MMR Status in RTK+ CRC


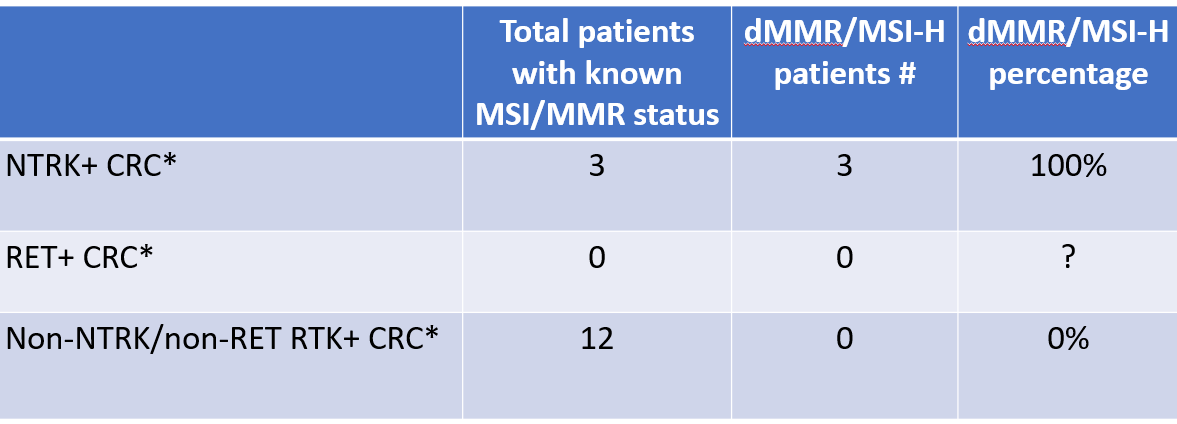


CRC: colorectal cancer. MSI: Microsatellite instability. MMR: Mismatch Repair. RTK: Receptor tyrosine kinase.

*1482 (10.0%) patients with CRC had their MSI or/and MMR status recorded in the database, which included 3 NTRK+, 3 EGFR+, 2 FGFR1+, 2 ERBB2+, 2 FLT1+, 1 FLT4+, 1 ALK+, and 1 PDGFRb+

**Supplementary Figures:**

**Figure S1**: Mean TMB in RTK+ and BRAF, KRAS mutated CRC

CRC: colorectal cancer. TMB: tumor mutation burden. RTK: Receptor tyrosine kinase.
